# Combating pancreatic cancer with PI3K pathway inhibitors in the era of personalised medicine

**DOI:** 10.1136/gutjnl-2018-316822

**Published:** 2018-11-05

**Authors:** James RW Conway, David Herrmann, TR Jeffry Evans, Jennifer P Morton, Paul Timpson

**Affiliations:** 1 Garvan Institute of Medical Research & The Kinghorn Cancer Centre, Cancer Division, Sydney, New South Wales, Australia; 2 St Vincent’s Clinical School, Faculty of Medicine, University of New South Wales, Sydney, New South Wales, Australia; 3 Cancer Department, Cancer Research UK Beatson Institute, Glasgow, UK; 4 Institute of Cancer Sciences, University of Glasgow, Glasgow, UK

**Keywords:** pancreatic cancer, clinical trials, cell biology

## Abstract

Pancreatic ductal adenocarcinoma (PDAC) is among the most deadly solid tumours. This is due to a generally late-stage diagnosis of a primarily treatment-refractory disease. Several large-scale sequencing and mass spectrometry approaches have identified key drivers of this disease and in doing so highlighted the vast heterogeneity of lower frequency mutations that make clinical trials of targeted agents in unselected patients increasingly futile. There is a clear need for improved biomarkers to guide effective targeted therapies, with biomarker-driven clinical trials for personalised medicine becoming increasingly common in several cancers. Interestingly, many of the aberrant signalling pathways in PDAC rely on downstream signal transduction through the mitogen-activated protein kinase and phosphoinositide 3-kinase (PI3K) pathways, which has led to the development of several approaches to target these key regulators, primarily as combination therapies. The following review discusses the trend of PDAC therapy towards molecular subtyping for biomarker-driven personalised therapies, highlighting the key pathways under investigation and their relationship to the PI3K pathway.

## Introduction

Accounting for ~95% of pancreatic cancers, pancreatic ductal adenocarcinoma (PDAC) has a very poor overall 5-year survival of 8% and is predicted to be the second leading cause of cancer-related deaths in the developed world by 2030.^1–3^ This has only marginally improved since the introduction of gemcitabine in 1995.^4 5^ Surgery remains the only curative treatment and is often applied with adjuvant chemotherapy, but as few as 10%–15% of patients are eligible at initial diagnosis.^6–9^ Most patients with PDAC have few or non-specific symptoms as the tumour develops, and this means that a large proportion are diagnosed at a late stage, already presenting with locally advanced or metastatic disease.^10^ For those patients that are not immediately eligible for resection, neoadjuvant chemotherapy can be given to reduce borderline tumours prior to resection.^11^ Recent clinical trials aimed at improving response to chemotherapy have demonstrated improved survival with patients treated with either a combination of gemcitabine and nab-paclitaxel or FOLFIRINOX (folinic acid, 5-fluorouracil, irinotecan, and oxaliplatin).^12–16^ However, patient tolerability may be limited with such aggressive treatment regimens.^16^ While improvements in surgical techniques and chemotherapy regimens are providing modest improvements in survival, there is a clear need to better understand this aggressive disease to facilitate both earlier diagnosis and elucidate new targets for combination therapies.

### PDAC progression model

**Figure 1:**
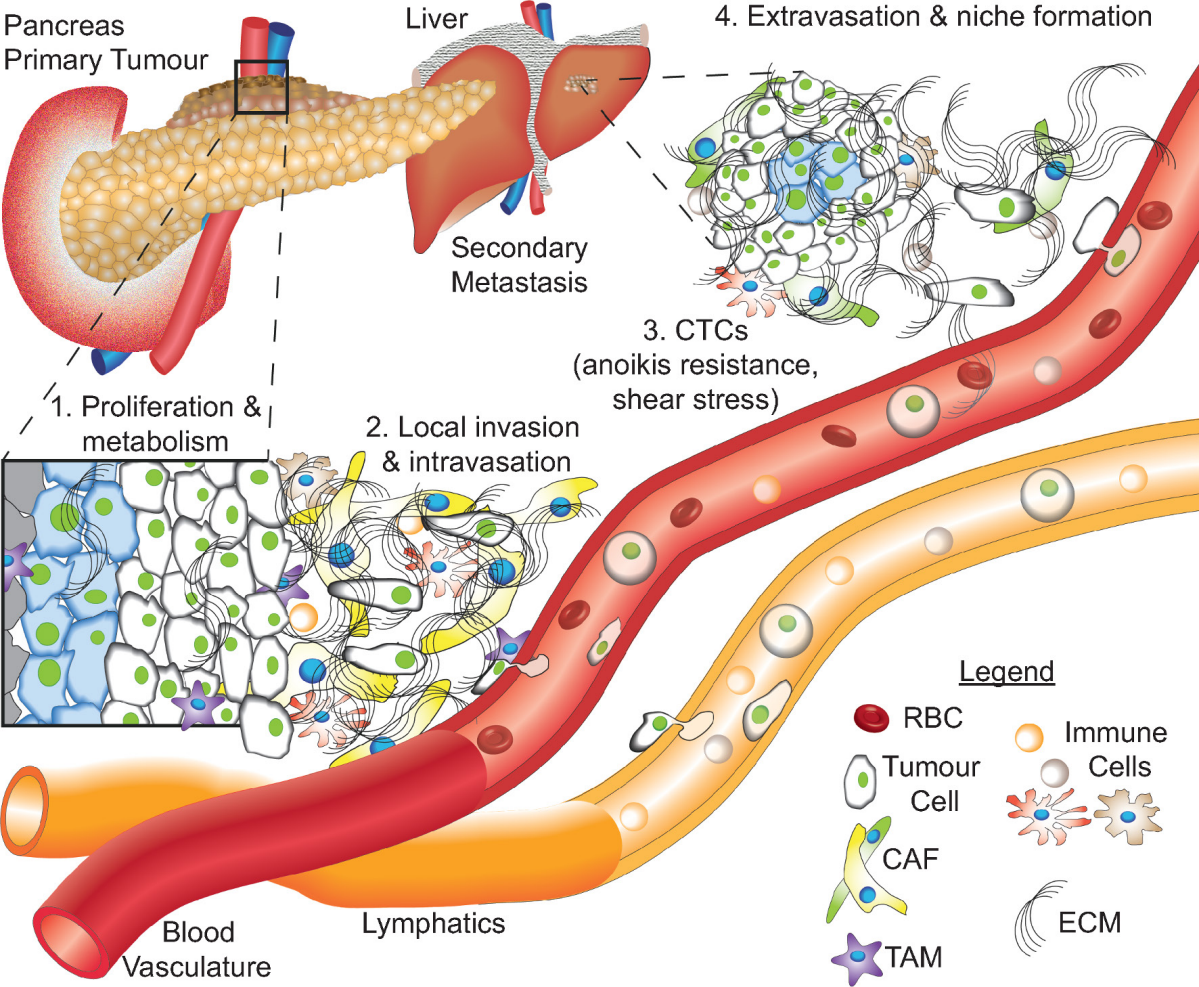
Schematic representation of PDAC progression from the primary tumour to a locally invasive disease and eventually metastasis. (1) Pancreatic cancer cells proliferate in the primary tumour, metabolising nutrients delivered by the blood vasculature and surrounding stroma. (2) Cancer cells invade through the extracellular matrix (ECM), including cancer-associated fibroblasts (CAFs) and tumour-associated macrophages (TAMs), among other cancer-associated cell types, eventually intravasating or invading into the lymph and travelling to distant sites. (3) Circulating tumour cells (CTCs) must develop resistance to anoikis, as well as shear stress, in order to survive in the circulation with red blood cells (RBCs) and leucocytes. (4) After travelling through the circulation, CTCs extravasate at secondary sites, commonly the liver, establishing a new niche. ECM, extracellular matrix; PDAC, pancreatic ductal adenocarcinoma.

The most widely accepted model for PDAC development is the progression model, in which PDAC originates from preinvasive pancreatic intraepithelial neoplasms (PanINs), which occur in distinct pathological stages, namely PanIN-1A, PanIN-1B, PanIN-2 and PanIN-3.^17–19^ In the early stages (ie, PanIN-1A and PanIN-1B or low-grade), a mucinous epithelium replaces the typically cuboidal morphology of normal pancreatic ducts, with a low level of dysplasia.^19–22^ Yet, recent work has suggested that pancreatic repair after injury, by the process of acinar-to-ductal metaplasia, may also be involved in PDAC initiation.^23–25^ As these PanINs progress (ie, to PanIN-2 and PanIN-3 or intermediate-grade and high-grade, respectively), dysplasia increases and is detectable as nuclear irregularity, loss of cell polarity and an increase in intraluminal apoptotic debris.^19–22^ This progression towards an invasive carcinoma has been shown to occur in parallel with increased proliferation and mutational burden from early preinvasive PanIN stages to metastatic PDAC (figure 1).^19 26^ Importantly, a mechanism underlying the switch from PanIN to metastatic PDAC remains unclear, but new genetically engineered mice that model multistep carcinogenesis may support the widely accepted stepwise mutational model, where some have suggested that catastrophic genomic events may instead trigger the transformation from preneoplastic lesions.^27 28^

A similar progression model has been proposed for intraductal papillary mucinous neoplasms (IPMNs), which are generally benign, but progress to an invasive carcinoma in up to 25% of cases.^29–32^ Both IPMNs and mucinous cystic neoplasms are radiologically detectable as macroscopic lesions and are classified according to the Sendai guidelines.^33^ They are typically distinguished from PDAC at a macroscopic level by mucoid contents and have distinct stromal subtypes at a microscopic level.^34 35^ Indeed, the mucoid expression itself has been used to subtype IPMNs according to whether the gene expression is gastric or intestinal, which clearly distinguished aggressive disease as the intestinal subtype.^35^

**Figure 2:**
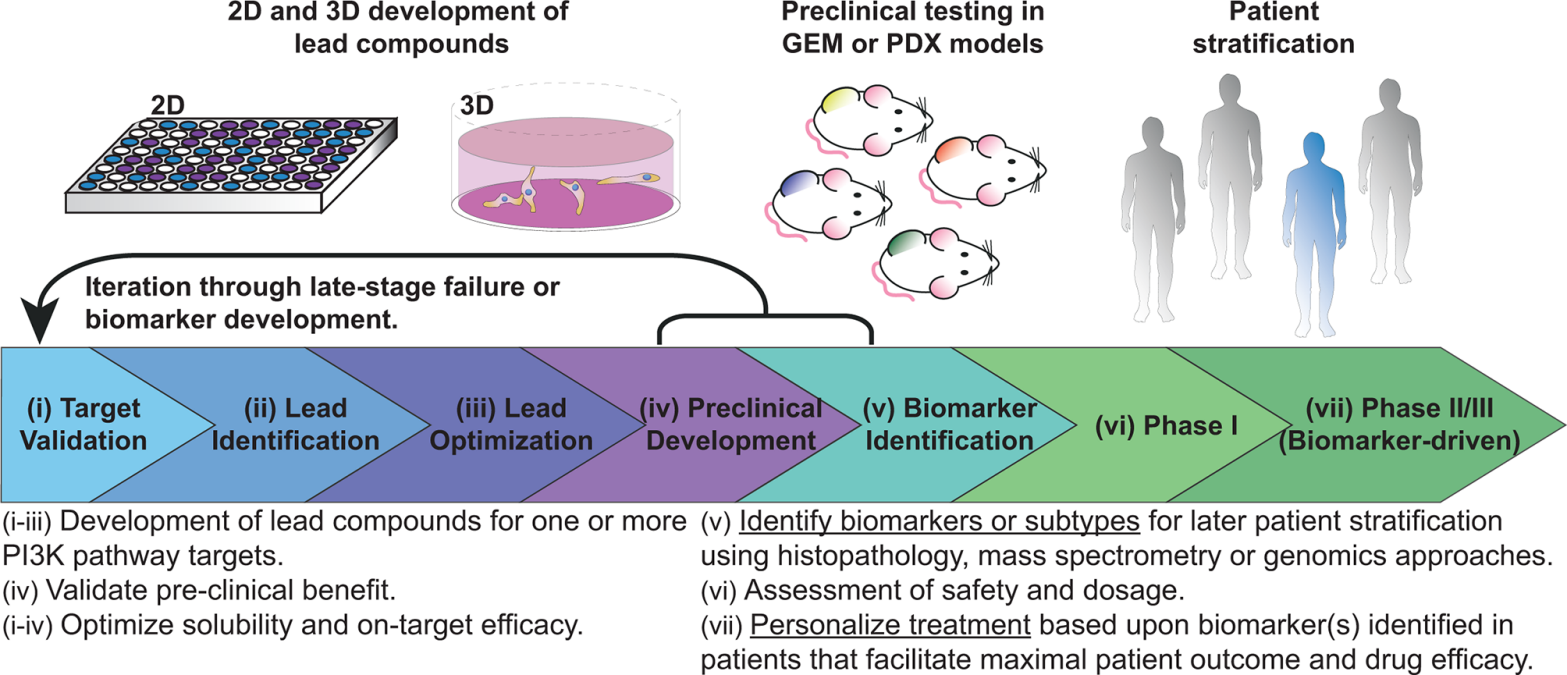
Adaptable drug development pipeline, demonstrating the progression of lead compounds through target validation, lead compound identification and optimisation, then preclinical validation. The necessary addition to this process is the identification of biomarkers to guide both lead compound development and later stratification in phase II/III clinical trials. These processes may be iterated to improve on-target efficacy, solubility and biomarkers. After safety and tolerability is confirmed in phase I clinical trials, biomarker-driven phase II/III may reduce the high attrition rates of lead compounds if appropriate patient stratification can demonstrate beneficial response in the assessed subsets of patients. These biomarkers may also provide opportunities for retrospective analysis and later iteration into clinical trials. PI3K, phosphoinositide 3-kinase.

Attempts have been made to classify PanINs in terms of their mutational burden. Initially, evidence of telomere shortening and mutations in *KRAS* were found to occur very early in PanIN progression.^36 37^ This excluded *KRAS* as a potential marker for PDAC progression but highlights the general classification as the earliest initiator mutation in PDAC, occurring in ~95% of PDAC cases.^26 38^ Progression through to PanIN-2 and PanIN-3 typically includes additional mutations in *TP53*, *SMAD4* and/or *CDKN2A*, but the vast molecular heterogeneity of this disease precludes any single mutation as essential for PDAC development.^38 39^ With this in mind, several large-scale sequencing and mass spectrometry approaches have been implemented to subtype the disease based on these molecular characteristics.^40–43^ The goal of such work is to better integrate biomarkers into the drug discovery pipeline, where lead compound development is performed hand in hand with biomarker identification (figure 2). This parallel preclinical development aims to foster a more personalised approach to clinical trial development, whereby each patient may be assessed for their respective molecular subtype and treatment is designed based on this result (figure 2).

### Molecular subtyping of PDAC

While several mutations occur at relatively high frequency in PDAC, mutations in the aforementioned genes are not currently associated with clinically actionable phenotypes. The milieu of lower frequency mutations, however, has motivated subtyping based on commonly mutated biological processes, termed gene programmes (GPs). The aim of such work is to develop therapeutic strategies that are selectively effective against specific tumour subtypes.^15 44^ Early work stratified PDAC according to an activated stromal index, which classified patients according to the ratio of alpha smooth muscle actin (immunohistochemical (IHC) staining) and collagen (stained with the collagen-specific Aniline blue).^45^ Such an index informs primarily on stromal targeting and alone is not sufficient to guide therapies aimed at complete tumour regression. Indeed, a second study took the opposite approach and removed the stroma by laser microdissection from the PDAC samples, prior to microarray analysis and subtyping of PDAC based on multivariate analysis of transcriptional profiles, namely classical, exocrine-like and quasimesenchymal (QM; table 1, see column ‘Collisson’).^40^ Such an approach allowed the authors to identify neoplastic epithelial-specific gene expression and to identify pathways involved in PDAC progression. This approach also motivated metabolite profiling within these subtypes, where classical tumours were shown to be lipogenic, while QM tumours were glycolytic.^46^ With clear subtype-specific metabolic targets, new avenues for combination therapies within a personalised setting are an obvious progression to improve patient responses. Additionally, increasing evidence for the importance of the stroma in disease progression means assessment of either the tumour or stroma in isolation is likely to be too simplistic to provide any lasting improvements in patient survival.^47^

**Table 1:** Molecular subtyping of patients with pancreatic cancer

	Collisson	Moffit	Bailey
Approach	Microarray	Microarray	Expression analysis (RNAseq and microarray)
Cohort	63 primary resected PDAC	145 primary resected and 61 metastatic PDAC tumours	96 RNAseq and 242 microarray primary patient samples
Tumour/stromal contribution	Microdissection	Multivariate analysis (virtual microdissection)	Macrodissection
Tumour subtypes	Classical	Classical	Pancreatic progenitor
			Immunogenic
	Exocrine-like		ADEX
	QM	Basal like	Squamous
Stromal subtypes	Not assessed	Activated	ESTIMATE
		Normal	

This pancreatic cancer subtype table is adapted from refs 40–42.

ADEX, Aberrantly Differentiated Endocrine eXocrine; ESTIMATE, Estimation of STromal and Immune cells in MAlignant Tumor tissues using Expression data; PDAC, pancreatic ductal adenocarcinoma; QM, quasimesenchymal.

Physical microdissection approaches rely on IHC to inform stromal activation state and also limit the application of patient subtyping by molecular approaches due to a low sample throughput and smaller sample volume.^48^ As large datasets become increasingly common, new analytical approaches improve the readouts incurred. A more recent approach to PDAC subtyping involved virtual microdissection of large microarray datasets, facilitating molecular subtyping of both the tumour and the stroma.^41^ Using multivariate analysis to distinguish tumour and stromal components, the tumour was split into a classical and more aggressive basal-like subtype, and the stroma was classified into activated or normal subtypes (table 1, see column ‘Moffit’). This additional stromal subtyping was also recently applied to PDAC patient-derived xenograft (PDX) tumours, whereby tumours classified as basal or classical were shown to have an ‘echo’ in the mouse stroma.^49^ They further demonstrated the power of their classifications through inhibition of cholesterol uptake in subtyped PDX models, where basal tumours were highly sensitive to inhibition, but classical tumours were shown to have higher *NPC1L1* expression and may require a greater concentration of inhibitor to achieve an equivalent growth inhibition.

Further subtyping was recently performed on a 328 primary patient PDAC cohort using expression analysis from RNAseq (96 patients) and microarrays (232 patients).^42^ This study included samples with invasive IPMN-associated PDACs and some metastatic tumours and, in contrast to the previous studies, applied macrodissection to excise areas of nonmalignant tissue, maintaining the stromal component in each sample.^38 42 50^ Tumour purity could then be inferred in terms of stromal and immune infiltration based on the Estimation of STromal and Immune cells in MAlignant Tumor tissues using Expression data approach.^51^ Beyond purity assessment, this approach facilitated assessment of GPs associated with microenvironmental factors, such as hypoxia, ECM deposition and activated immune pathways.^42^ The microenvironmental influence on cancer progression is an essential consideration for emerging therapies, where immune cells, cancer-associated fibroblasts and ECM components are regularly associated with cancer progression (figure 1). Inclusion of this stromal contribution, as well as the large breadth of patient samples, allowed the authors to reclassify PDAC into four distinct subtypes (summarised in table 1 (see column ‘Bailey’) and box 1). This is particularly important in light of the high attrition rates for lead compounds currently experienced by the pharmaceutical industry, where more detailed molecular analysis prior to treatment is expected to improve both patient and trial outcomes (figure 2).^52–54^

Box 1 **Pancreatic cancer subtypes**

**Molecular subtypes (described in ref 42)**

*Immunogenic*

Patients were classified with this subtype if they showed evidence of high levels of immune infiltrate, which presents an inferred opportunity for emerging immunotherapies.

*Pancreatic progenitor*

This subtype shares several gene programmes (GPs) with the immunogenic subtype. It is defined by an increased activity of transcriptional networks associated with pancreatic endodermal cell fate. Pancreatic ductal adenocarcinoma (PDAC) associated with IPMN typically fell within this subtype, where an increase in fatty acid metabolism and O-linked glycosylation of mucins was upregulated.

*Aberrantly Differentiated Endocrine eXocrine*

This subtype presented with higher activity of GPs associated with exocrine secretion, consistent with a more differentiated pancreatic lineage.

*Squamous*

Describing the most aggressive PDAC tumours, including the KPC GEM PDAC model, this subtype is defined by an upregulation of GPs associated with hypoxia, metabolic reprogramming, ECM deposition, squamous differentiation and proliferation.

**Neoantigen quality (described in ref 280)**

Antigens encoded by tumour-specific genes (neoantigens) are enriched in long-term PDAC survivors, along with high T cell infiltration. The quality of neoantigen may then provide a biomarker for emerging immunotherapies.

**Mass spectrometry subtypes (described in ref 43)**

Using 8 or 33 phosphosites as classifiers, the Australian Pancreatic Cancer Genome Initiative patient-derived cell lines or commercially available pancreatic cancer lines from the American Type Culture Collection were grouped into three subtypes based on their pTyr levels. Of these, subtype 3 in both cell line cohorts was enriched for receptor tyrosine kinase (RTK) phosphorylation and showed increased sensitivity to the epidermal growth factor receptor inhibitor erlotinib. This suggests that mass spectrometry approaches may provide a binary system for classifying patients into RTK or phosphoinositide 3-kinase pathway targeted therapies.

The goal of this molecular phenotyping is to establish trials, such as IMPaCT, PRECISION-Panc, SHIVA, or biomarker-driven avatar trials (NCT02795650), where actionable molecular data guides therapies.^55 56^ These trials have established the feasibility of biopsy collection for pancreatic cancer within a clinical setting, where molecular assessment was performed by IHC or genomic approaches. However, biomarker-driven trials for pancreatic cancer remain infrequent, despite increasing evidence for a lack of stratification leading to late-stage failure. This is particularly evident for PI3K pathway inhibitors, where preclinical efficacy is driving their assessment in a clinical setting, but biomarker-driven trials in pancreatic cancer are sorely lacking (table 2). This is in stark contrast to the increase in biomarker-driven trials in other cancers, where biomarkers such as loss of phosphatase and tensin homolog (PTEN), *PIK3CA* mutation or Akt amplification/mutation are increasingly used to stratify patients for treatment with PI3K pathway inhibitors.^57^ Further to this goal, the subtyping approaches described above may also provide novel clinically actionable biomarkers or GPs to allow patient-selective assessment of PI3K pathway inhibitors to push PDAC survival beyond the current standard of care.

**Table 2:** List of PI3K pathway inhibitors currently undergoing clinical development for pancreatic cancer

Target	Inhibitor	Phase	Status	Patients	Combination	NIH number	Reference(s)
Akt inhibitors							
Pan-Akt	MK2206	I	Completed	AdvST/MST (~9% pancreatic cancer)	Monotherapy	NCT00670488	262 263
		I	Completed	AdvST/MST	Selumitinib (MEKi)	NCT01021748	
		I	Completed	PDAC (PTEN loss)	Monotherapy	NCT00848718	264
		I	Completed	Pancreatic cancer	Dinaciclib (CDKi)	NCT01783171	
		II	Completed	Pancreatic cancer	Selumitinib (MEKi) versus mFOLFOX6	NCT01658943	265
	Afuresertib (GSK2110183)	I	Completed	AdvST (21% pancreatic cancer)	Trametinib (MEKi)	NCT01476137	266
		II	Ongoing	AdvST		NCT01531894	
	Uprosertib (GSK2141795)	I	Completed	Pancreatic cancer	Trametinib (MEKi)	NCT01138085	
		I	Completed	AdvST		NCT00920257	
	Oleandrin (PBI-05204)	I	Completed	AdvST (6% pancreatic cancer)		NCT00554268	267
		II	Ongoing	Metastatic pancreatic cancer		NCT02329717	
	Perifosine	II	Completed	Locally advanced or metastatic pancreatic cancer		NCT00053924	
		II	Completed	Locally advanced or metastatic pancreatic cancer		NCT00059982	268
	RX-0201	II	Completed	Metastatic pancreatic cancer	Gemcitabine	NCT01028495	
Rapalogs							
mTORC1 (FKBP12)	Sirolimus (rapamycin)	I	Completed	Pancreatic cancer	Sunitinib (RTKi)	NCT00583063	150
		I	Completed	Pancreatic cancer	Sorafenib (RTKi)	NCT00449280	150
		II	Completed	Pancreatic cancer		NCT00499486	
		II	Completed	Pancreatic cancer		NCT00276744	
		I/II	Ongoing	PDAC	Metformin	NCT02048384	
		I	Ongoing	Pancreatic cancer	Vismodegib (SMOi)	NCT01537107	
	Temsirolimus *(CCI-*779, Torisel)	I	Completed	Pancreatic cancer	Lenalidomide	NCT01183663	
		I	Terminated	PDAC	Gemcitabine	NCT00593008	
		I/II	Ongoing	Pancreatic cancer	Nivolumab (PD-1i)	NCT02423954	
		II	Completed	Locally advanced or metastatic pancreatic cancer		NCT00075647	95
	Everolimus (RAD001)	I	Completed	Pancreatic cancer	Sorafenib (RTKi)	NCT00981162	
		I	Completed	Pancreatic cancer	Trametinib (MEKi)	NCT00955773	269
		I/II	Completed	PDAC	Gemcitabine	NCT00560963	
		I/II	Completed	Pancreatic cancer	Cetuximab (EGFRi) and capecitabine	NCT01077986	99
		II	Terminated	Pancreatic cancer	Erlotinib (EGFRi)	NCT00640978	95
		II	Completed	Pancreatic cancer		NCT00409292	94
		I/II	Recruiting	PDAC	Ribociclib (CDKi)	NCT02985125	
	Ridafirolimus	I	Completed	AdvST (12% pancreatic cancer)	Bevacizumab (VEGFRi)	NCT00781846	149
PI3K inhibitors							
PI3K isoform p110α	Alpelisib (BYL719)	I	Ongoing	Pancreatic cancer	Gemcitabine and abraxane	NCT02155088	
Pan-PI3K	Buparlisib (BKM120)	I	Completed	Pancreatic cancer	mFOLFOX6	NCT01571024	270
		I	Completed	Pancreatic cancer	LDE225 (SMOi)	NCT01576666	
		I	Completed	Pancreatic cancer	Trametinib (MEKi)	NCT01155453	271
		I	Ongoing	AdvST	MEK163 (MEKi)	NCT01363232	
	PX-866	I	Completed	AdvST (5% PDAC)	Docetaxel	NCT01204099	272
	ZSTK474	I	Completed	AdvST		NCT01280487	
	Copanlisib (BAY 80–6946)	I	Completed	AdvST (18% pancreatic cancer)		NCT00962611	273
Dual PI3K pathway inhibitors							
mTORC1/2	Vistusertib (AZD2014)	I	Completed	AdvST		NCT01026402	98
		II	Recruiting	AdvST (RICTOR amplified)		NCT03166904	
		II	Recruiting	AdvST: combination with Selumitinib (MEKi)		NCT02583542	
		II	Recruiting	AdvST (TSC1/2 loss or mutation)		NCT03166176	
		II	Recruiting	AdvST: combination with Olaparib (PARPi)		NCT02576444	
p70-S6K and Akt	LY2780301	I	Complete	AdvST (~22% pancreatic cancer)		NCT01115751	274
PI3K and mTOR	Dactolisib (NVP-BEZ235)	I	Completed	AdvST	MEK162 (MEKi)	NCT01337765	
	NVP*-*BGT226	I	Completed	AdvST (2% pancreatic cancer)		NCT00600275	275
	Voxtalisib (SAR245409, *XL765*)	I	Completed	AdvST (4% pancreatic cancer)		NCT00485719	276
	SF1126 (LY294002 prodrug)	I	Completed	AdvST (5% pancreatic cancer)		NCT00907205	277
	Gedatolisib (PF-05212384, PKI-587)	I	Terminated	AdvST (5% pancreatic cancer)	Irinotecan	NCT01347866	278
		I	Completed	AdvST (4% PDAC)		NCT00940498	279
		I	Recruiting	AdvST	Palbociclib (CDKi)	NCT03065062	

AdvST, advanced solid tumours (including pancreatic cancer); CDKi, cyclin-dependent kinase inhibitor; EGFR, epidermal growth factor receptor; EGFRi, EGFR inhibitor; MEKi, MAPK/ERK kinase inhibitor inhibitor; mFOLFOX6, modified FOLFOX (ie, 5-fluorouracil and oxaliplatin); MST, metastatic solid tumours (including pancreatic cancer); mTOR, mechanistic target of rapamycin; NIH, National Institutes of Health; PARP, poly (ADP-ribose) polymerase; PARPi, PARP inhibitors; PDAC, pancreatic ductal adenocarcinoma; PD-1, programmed death-1; PD-1i, PD-1 inhibitor; PI3K, phosphoinositide 3-kinase; RTKi, receptor tyrosine kinase inhibitor; SMOi, smoothened inhibitor; VEGFRi, vascular endothelial growth factor receptor inhibitor.

## The phosphoinositide 3-kinase (PI3K) pathway

A broad range of cancer types, including pancreatic cancer, have been candidates for targeting of the PI3K pathway, due to amplification, mutation or loss of key regulators.^58 59^ The PI3K pathway mediates transduction of signals from both extracellular and intracellular sources, including growth factors and nutrients, leading to downstream signalling involved in cancer growth, survival and progression (figure 1).^58 60 61^ The pathway is also essential for many cancer-associated activities, including endothelial cell sprouting for angiogenesis, macrophage transcriptional reprogramming, T cell differentiation and homeostasis and fibroblast-supported chemoresistance (figure 1).^62–65^ Collectively, this suggests that application of PI3K pathway inhibitors as a PDAC therapy may provide an opportunity for dual targeting of cancer cells and the deregulated cancer-associated stromal components.

PDAC is regularly associated with increased Akt activity, which has been identified in ~60% of PDAC samples, with amplification of the *AKT2* oncogene occurring in 10%–20% of PDAC cases.^66–68^ Akt is a key effector of the PI3K pathway, downstream of both PI3K and receptor tyrosine kinases (RTKs; table 2). Furthermore, PDAC tumours have been shown to bear an activating mutation in *PIK3CA* and/or loss of the tumour suppressor PTEN in ~4% and 25%–70% of cases, respectively.^50 69–72^ Interestingly, patients with low PTEN expression have a much higher incidence of recurrence or metastasis, compared with those with high PTEN.^72^ Furthermore, it has been shown that PDAC patients with high PI3K pathway activity show a significantly poorer survival than those with low activation of this pathway.^73^

**Figure 3:**
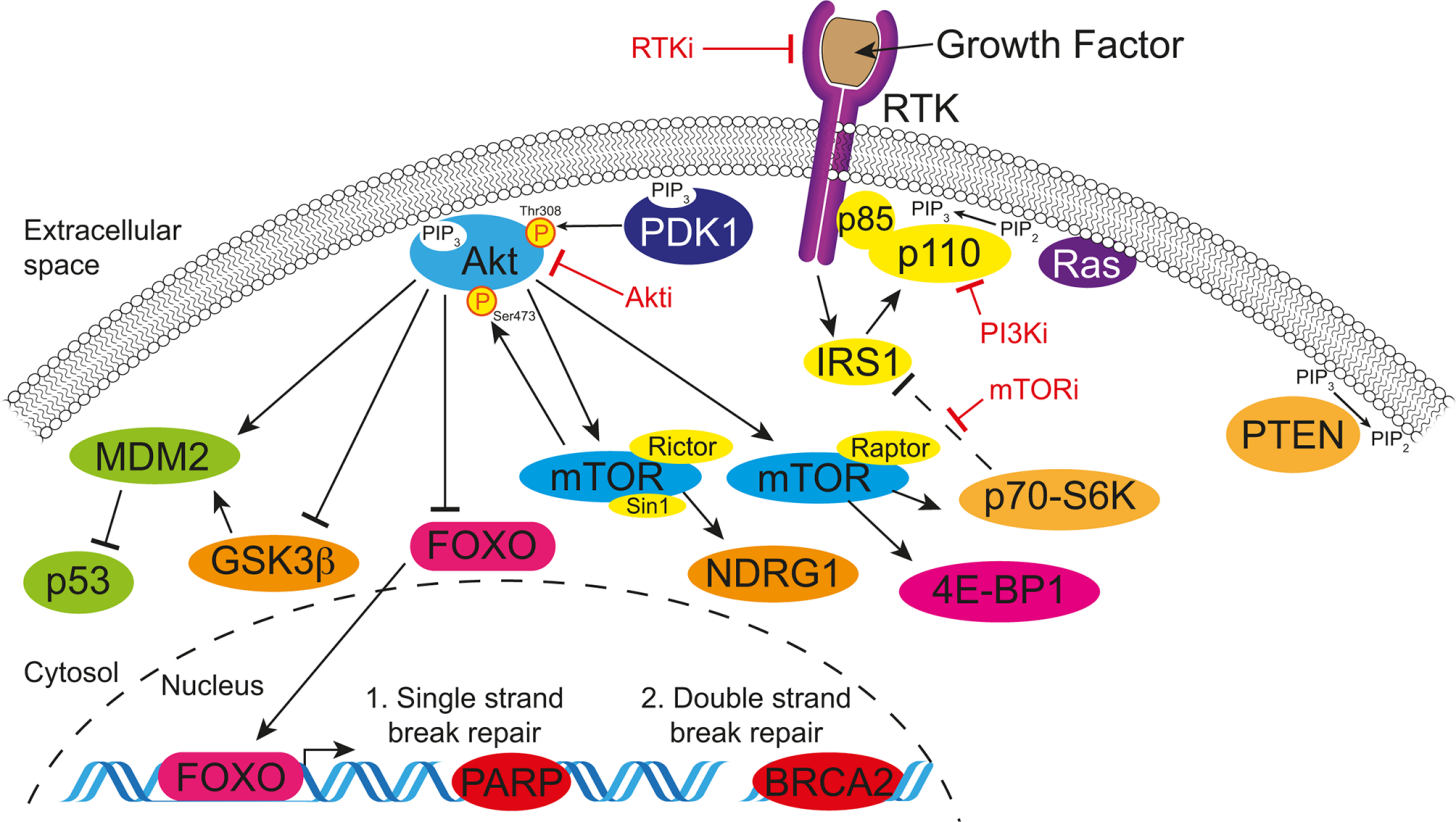
Simplified schematic of the PI3K pathway, which highlights the common targets for small molecule inhibitors. Briefly, signalling from growth factors activates RTKs and recruits PI3K and other scaffold proteins to the cell membrane, where PIP_2_ is converted to PIP_3_. This recruits phosphoinositide-dependent kinase-1 (PDK1) and Akt to the membrane and leads to downstream signalling through the kinase activities of Akt. (1) Single-strand break repair is regulated primarily by PARP and inhibition of PARP can lead to genomic instability. (2) Double-stranded break repair is primarily regulated by a complex with BRCA2, which is lost in familial pancreatic cancer and some PDAC cases and can lead to genomic instability. Genomically unstable tumours require the PI3K pathway to maintain survival pathways and PI3K pathway inhibition may be an emerging option for patients with BRCA2 mutations or in combination with PARP inhibitors. More exhaustive pathway maps can be found in refs 61 80. P13K, phosphoinositide 3-kinase; RTKs, receptor tyrosine kinases.

Several signalling pathways are known to converge on the MAPK and PI3K pathways as effectors of cellular response within the cell. For example, in ~95% of cases, pancreatic cancer is driven by activating mutations in *KRAS*, which in turn activates PI3K signalling through the p110α subunit, along with another pathway component PDK1, indicating that a large proportion of patients could benefit from effective targeting of this pathway (figure 3).^28 73–76^ Furthermore, detection of mutations in *PIK3CA* can be predictive for improved patient response in preclinical models of PDAC and in patients with breast cancer stratified according to detection of mutations in circulating cell-free DNA.^74 77–79^ Given the varied roles of different PI3K isoforms in both the tumour and associated stromal cells, isoform-specific inhibitors provide isolated targeting of oncogenic signalling and allow redundancy to alleviate off-target side effects in healthy tissues (table 2; reviewed in refs 80 81). Notably, a PI3Kα-specific inhibitor has shown promising efficacy in combination with an EGFRi in PDAC with high EGFR and Akt phosphorylation.^82^ Interestingly, PIK3CA mutations in breast cancer have also been linked with Akt-independent tumour progression through SGK3 and highlight the importance of all levels of this key signalling cascade.^83^ Similarly, isoform-specific PI3Kβ inhibition extended PDAC survival beyond mTORC1/2 targeting alone,^84^ and in other cancers, inhibition of PI3Kβ and PI3Kδ has shown antimetastatic effects and suggests a role of PI3K in tumour metastatic dissemination.^85 86^ Furthermore, isoform-specific inhibition of PI3Kδ in cancer-associated immune cells was shown to downregulate their tolerance to PDAC, which improved the activity of T cells against the cancer.^87^ Collectively, we see strong evidence accumulating for the efficacy of upstream isoform-specific targeting of PI3K in emerging PDAC combination therapies.

Concordantly, evidence for the validity of downstream pathway targeting is highlighted in a genetically engineered mouse model of mutant *Kras^G12D^*-driven PDAC, which was applied in concert with a sleeping beauty transposon library, both conditionally expressed (ie, *LSL-Kras^G12D^* and *LSL-SB11*) under pancreas-specific *Pdx1-Cre*.^88 89^ These approaches identified several genes within the MAPK and PI3K pathways as cooperating mutations for *Kras^G12D^*-driven PDAC. Similarly, recent assessment of kinases with the highest levels of absolute and differential expression in a panel of pancreatic cancer cell lines demonstrated significantly reduced cell number after knockdown of EGFR, Akt2, PLK2 or MET.^90^ This review will focus on PI3K pathway targeting in PDAC (see also table 2, PI3K pathway inhibitors under clinical investigation in PDAC).

### PI3K pathway inhibitors in the clinic

After the discovery and isolation of rapamycin on the island of Rapa Nui from *Streptomyces hygroscopicus*, over 30 years of research continues to find new therapeutic applications for this compound.^91^ For example, the mTOR inhibitor (mTORi) rapamycin was recently assessed in PDAC driven by activated PI3K/AKT signalling via PTEN loss, where targeting of mTORC1 by rapamycin significantly reduced the onset and progression of the disease (figure 3).^92^ Work to improve the solubility and bioavailability of rapamycin-based compounds (rapalogs) has seen modification at the C-42 position through addition of an ester, ether or phosphonate group to generate temsirolimus, everolimus and ridaforolimus, respectively.^60^ Clinical trials in pancreatic neuroendocrine tumours (pNETs) have demonstrated a clear benefit for rapalogs as single agents (table 2);^93^ however, no significant improvements have been identified for rapalogs as single agents in PDAC.^94 95^ This has been attributed to an upstream feedback loop where inhibition of mTORC1 alone relieves the inhibitory phosphorylation of insulin receptor substrate 1 (IRS1)by p70-S6K and mTORC1, leading to an upregulation of Akt phosphorylation (figure 3).^96 97^ Hence, while trials of rapalogs may benefit from stratification for patients with high PI3K pathway activity, newer agents that target both mTORC1 and mTORC2, or other pathway components, allow the negation of this feedback loop with promising therapeutic potential (table 2).^84 98^ Importantly, new combination therapies with rapalogs should consider the combined toxicity with other targeted compounds. For example, combination of everolimus with the RTKi cetuximab was found to be too toxic for patients with PDAC in a phase I/II clinical trial, while the single agents show minimal toxicity.^94 99 100^ With this in mind, trials are still ongoing in PDAC using rapalogs in combination therapies (table 2).

Next-generation dual PI3K pathway inhibitors are being developed that take advantage of the homology of the kinase domains from class I, II and III PI3Ks and those of phosphoinositide 3-kinase-related kinases, such as mTOR, ATM and DNA-PK (figure 3, table 2).^101^ However, these dual inhibitors have been linked with drug-related dosage-dependent toxicities, such as hyperglycaemia, nausea, vomiting and diarrhoea, consistent with PI3K isoform targeting, and reinforce the need for preclinical assessment of the additive or synergistic toxicities when developing novel combination therapies.^81^ Moving forward, improvements in solubility are driving greater oral bioavailability, where lower drug dosages can show equivalent drug efficacy and gastrointestinal toxicities are readily reduced.^102 103^ One exciting example of a dual PI3K pathway inhibitor is AZD2014, which was developed by iterative structure–activity relationship medicinal chemistry approaches to have high aqueous solubility and a potent inhibitory effect against both mTORC1 and mTORC2.^104^ Recent preclinical work by our group has demonstrated potent antiproliferative and anti-invasive effects in the KPC (*LSL-Kras^G12D^*, *LSL-Trp53^R172H^* and *Pdx1-Cre*) GEM PDAC model^84 105^ and after a promising phase I clinical trial in advanced solid tumours (AdvSTs), AZD2014 has progressed to phase II biomarker-driven clinical trials either alone or in combination with a MAPK/ERK kinase inhibitor (MEKi) (table 2).^98^ The additional anti-invasive role for AZD2014 is consistent with increasing evidence for the emerging antimetastatic and anti-invasive effect of PI3K pathway targeting (figure 1).^105–109^ Indeed, the opposing roles for the different Akt isoforms in cell motility have identified an invasion and metastasis promoting role of Akt2 but an inhibitory role for Akt1.^110–114^ The dual role of Akt1 in either promoting tumour growth or metastasis was recently shown to be regulated by the inositol polyphosphate 5-phosphatase (PIPP), where PIPP ablation resulted in reduced metastasis but increased tumour growth.^108^ Similarly, mTORC1 and mTORC2 have been shown to regulate migration and invasion through Rac1 and RhoA.^58 80 115^ Furthermore, mTOR inhibition dramatically reduced metastasis in prostate cancer, highlighting the broader potential of PI3K pathway therapeutics as antimetastatic agents.^116^ Intriguingly, the mTOR inhibitor, everolimus, resulted in a partial response in a patient with pancreatic cancer that was induced by Peutz-Jeghers syndrome (PJS).^117^ PJS is caused by a tumour-suppressor gene mutation in the serine threonine kinase 11 gene (*STK11*, also known as *LKB1*), which results in ~11%–36% of patients with PJS developing pancreatic cancer.^117^ This loss of STK11 leads to a loss of suppression of mTOR signalling and raises the tantalising possibility that mTOR inhibition could have monotherapy efficacy in PDAC in selected cases with a similar genetic background.

## Emerging opportunities for combination therapies

### Opportunities for patients with RTK amplification or mutation

The RTK family comprises several subfamilies that are not limited to ErbB, fibroblast growth factor receptors (FGFRs), insulin and insulin-like growth factor receptors, platelet-derived growth factor receptor (PDGFR), vascular endothelial growth factor (VEGF) receptor (VEGFR) and Axl and the Ephrin receptors. Inhibition of these receptors using RTK inhibitors (RTKi) generally takes one of three forms: antibody or recombinant protein inhibition of the extracellular ligand binding domain, inhibition of the ligand itself, or targeting of the cytoplasmic tyrosine kinase domain.

In recent work, 19 PDAC cell lines from the American Type Culture Collection and 17 patient-derived cell lines (PDCLs) from the Australian Pancreatic Cancer Genome Initiative collection, sequenced as part of the International Cancer Genome Consortium (ICGC), were used to assess global phosphotyrosine (pTyr) profiles in PDAC by mass spectrometry.^43^ This approach allowed the authors to define two sets of classifier mutations (8 and 33 pTyr sites) that predicted three PDAC subtypes over the two cell line panels (box 1). Interestingly, when RTK activity was enriched, the cell lines showed an enhanced sensitivity to the EGFRi erlotinib, suggesting that this subtyping approach may provide a method to stratify patients for RTK-targeted therapies. While the somatic mutation profiles did not correlate with the pTyr-based subtyping, similar GPs were identified between both the genomic and mass spectrometry studies.^42 43^ However, at the mass spectrometry level, it was possible to identify the activation status of kinase networks and receptors. This lends weight to the overlapping use of both genomic and mass spectrometry approaches to assess aberrant pathway expression, mutation status and, importantly, activation state and provides clear motivation for the incorporation of both techniques into the drug discovery pipeline (figure 2).

#### The ErbB family

RTKs are transmembrane receptors that communicate signals from ligands outside of the cell by activating their cytoplasmic tyrosine kinase domains, which facilitate downstream signalling within the cell, typically through activation of the MAPK and PI3K pathways. The ErbB family contains four RTKs structurally related to the epidermal growth factor receptor (EGFR; human epidermal growth factor receptor (HER) 1 and ErbB-1). EGFR expression is observed in normal pancreatic ducts but has been shown to increase from the early stages of PanIN development through to PDAC.^118–120^ Targeting of the EGFR receptor with the small molecule inhibitor erlotinib in combination with gemcitabine resulted in a statistically significant, but clinically modest, improvement in overall survival compared with gemcitabine monotherapy in patients with metastatic disease and has also been evaluated in the adjuvant setting.^121 122^ These studies subsequently motivated the assessment of predictive markers that would stratify patients for this treatment.^123–126^ These studies found conflicting evidence for *KRAS* mutational status as a predictive or prognostic marker for erlotinib response but suggested that mutations or amplification of EGFR may be sufficient to stratify patients for therapy. Interestingly, expression of ErbB-3 (HER3) has been associated with sensitivity to erlotinib treatment in pancreatic cancer cell lines and therefore may prove an effective biomarker for adjuvant erlotinib for patients with PDAC.^127–129^ ErbB-3 requires heterodimerisation for downstream signalling through the PI3K pathway and expression in PDAC is a poor prognostic factor for survival.^127–129^ Another emerging personalised approach to PDAC therapy comes from the success of targeting ErbB-2 (neu and HER2) amplified tumours with a humanised monoclonal antibody.^130^ ErbB-2 amplification in PDAC has a relatively low prevalence of 2%;^50 131 132^ however, clinical trials with trastuzumab (Herceptin) in combination with chemotherapy have shown beneficial responses in metastatic PDAC patients with ErbB-2 amplification,^133 134^ and studies are still ongoing in metastatic or recurrent PDAC.^56^ ErbB-4 (HER4) is the last member of the ErbB family but is only weakly expressed in PDAC.^135 136^ However, given the established importance of the other ErbB family members in PDAC progression, they may also prove effective biomarkers for inhibition of the PI3K pathway, which is less sensitive to changes in receptor dimerisation.

#### FGFR, PDGFR and VEGFR stromal targeted therapies and biomarkers

The FGFR, PDGFR and VEGFR families share sufficient structural homology that targeting of these receptors often has overlapping responses. In PDAC, overactivation of FGFR signalling has been associated with 2% of patients, and targeting of this receptor in PDAC using dovitinib has recently completed a phase I clinical trial in combination with chemotherapy, after a promising preclinical study, where dovitinib was found to exert its effect through decreased Akt activity (NCT01497392).^50 137^ Furthermore, FGFR and PDGFR upregulation in a proof-of-principle study using Kras-deficient PDAC was recently linked with increased sensitivity to PI3K pathway targeting highlighting the essential supportive role this pathway plays in PDAC progression and the potential of RTKs as biomarkers for patient stratification.^138^ Interestingly, inhibition of FGFR alone or in combination with PDGFR inhibition was not sufficient to decrease cancer cell proliferation to the same degree as PI3K pathway inhibitors, indicating that multiple RTK pathways feed into PI3K activation in PDAC and that PI3K inhibition may provide an opportunity for targeting of multiple de-regulated RTK pathways simultaneously (figure 3).^138^

Due to the highly desmoplastic reaction characteristic of PDAC, it is important to consider the stromal responses to therapies and even look for new targets within this compartment. Moreover, the effect of FGFR targeting in stromal pancreatic stellate cells has also demonstrated a beneficial outcome by reducing cancer cell invasion and hence better containing the tumour.^139^ This suggests that PI3K pathway inhibition may also have an antistromal effect that reduces the protumourigenic role of the activated cancer-associated fibroblasts and stellate cells, but as yet, this effect has not been assessed. Interestingly, overexpression of FGFR has also been used in a less conventional approach, where targeting this cell-surface receptor with an antibody-conjugated adenovirus specifically delivered a viral gene.^140^ This viral gene then predisposed these cells to antiviral therapy by ganciclovir. While this work has not progressed beyond preclinical models, other alternative therapies, such as antibody-conjugated nanoparticles, toxins, viruses or CAR-Ts,^141–144^ highlight the variety of emerging therapies that could potentially combat this primarily treatment-refractory disease.

PDGFR is less commonly mutated in PDAC, but upregulation of PDGFR signalling has been implicated as a mechanism for metastatic progression in p53-mutated tumours.^42 145 146^ Interestingly, one patient with PDAC who responded well to AZD2014 therapy in a phase I trial was found to have a PDGFR1A mutation, and this may present a novel biomarker for therapies aimed at PI3K pathway inhibition.^98^ An important function of PDGFR signalling is an overlapping role with VEGFR signalling for angiogenesis, which has been extensively assessed as a target in PDAC.^147^ After promising clinical trials led to approval of the mTORC1 inhibitor rapamycin and the broad-spectrum RTKi Sunitinib in pNETs, which are typically highly vascularised, several clinical trials began looking at the effectiveness of these inhibitors in PDAC (table 2).^93 148^ However, the antiangiogenic effects in PDAC provided minimal clinical benefit and future clinical trials are looking at the application of RTKi as part of combination therapies (table 2).^149–152^ One interesting target that has emerged from VEGFR targeting strategies is the discovery that placental growth factor, a VEGF homologue, is specifically upregulated in tumour vasculature and provides a target for disease-specific angiogenesis, without affecting normal healthy vessels.^153^ However, the effectiveness of this strategy remains controversial and has yet to progress to the clinic.^154^

#### Ephrin receptors as predictive biomarkers or novel targets

The largest known RTK family is that of the Ephrin receptors, of which both the EphrinA and EphrinB subfamilies are associated with poorer survival in patients with PDAC and are predictive of tumour proliferative and growth capacity.^155 156^ Indeed, increased activity of EphrinA2 has been associated with *Kras-*driven PDAC progression and knockdown in a mouse model of PDAC decreased metastasis.^43 157^ Furthermore, axon guidance GPs in which EphrinA5 and EphrinA7 play a role have been implicated in PDAC development, providing further motivation for application of the EphrinA/EphrinB receptors as predictive biomarkers for aggressive disease.^38^ Their continued association with PDAC has led to several approaches to therapeutically target these receptors.^158^ For example, a recent toxin-conjugated monoclonal antibody against the EphrinA2 receptor MEDI-547 completed phase I clinical trials in treatment-refractory solid tumours.^159^ Similarly, the broad-spectrum small molecule tyrosine kinase inhibitor dasatinib has an established inhibitory effect on the intracellular kinase domains of Ephrin receptors and provides a parallel approach for targeting of other RTKs.^160^ After promising preclinical studies, dasatinib has progressed to clinical trials for metastatic PDAC in combination with FOLFOX (NCT01652976) or gemcitabine/erlotinib (NCT01660971) chemotherapy.^161–163^ However, dasatinib in combination with gemcitabine did not improve overall compared with gemcitabine and placebo in locally advanced, non-metastatic PDAC.^164^ Another common approach to target upregulated Ephrin signalling is to inhibit the downstream pathways, such as the MAPK or PI3K pathways.^158^ Importantly, as PDAC therapy necessarily turns towards predictive biomarkers to guide personalised therapies, upregulation of Ephrin family members may predict response to RTK, MAPK or PI3K pathway inhibition in PDAC.

### Canonical and non-canonical inhibition of aberrant transforming growth factor β (TGFβ) signalling

The membrane-bound TGFβ receptor is mutated at a relatively low frequency in PDAC.^50 70^ However, disruptions in other pathway components occur in ~47% of patients, including mutations in *SMAD4*, *SMAD3*, *TGFBR1*, *TFGBR2*, *ACVR1B* and *ACVR2A*.^42^ There is a complex relationship between TGFβ signalling and either tumour suppression or metastatic spread.^165^ Indeed, loss of *SMAD4* is indicative of a poorer prognosis, while TGFβ pathway activation is associated with an epithelial-to-mesenchymal transition, one of the driving factors for metastatic dissemination.^165 166^ This has made TGFβ signalling the focus of recent clinical trials combining TGFβ receptor inhibition with gemcitabine (NCT02154646 and NCT01373164) or immunotherapy (NCT02734160). However, these trials are not biomarker driven and hence are not stratified for *SMAD4* mutational status, which is associated with failure of adjuvant chemotherapies in PDAC.^167 168^ The role of *SMAD4* in TGFβ signalling is primarily tumour suppressive, and this function may limit application of TGFβ receptor inhibitors, where they would best be applied to patients with *SMAD4* deletion.^169^ A key non-canonical mediator of TGFβ signalling is the PI3K pathway, which was shown to be inhibited by TGFβ receptor inhibitors and activated by endogenous TGFβ.^165 170 171^ Hence, an alternative route, independent of the tumour-suppressive functions of TGFβ signalling, may be through inhibition of these non-canonical signalling pathways.

### Targeting DNA damage repair defective tumours

Aberration in DNA damage repair pathways, such as mutations in *BRCA1*, *BRCA2*, *PALB2* or *ATM*, are commonly associated with increased risk of familial pancreatic cancer,^172 173^ but also occur in the later stages of PanIN development and PDAC.^18 20 42 56^ Loss of these DNA repair proteins leads to genomic instability and predisposes patients to breast, ovarian, prostate and pancreatic cancers.^174–176^ Patients with mutations in this pathway in other cancers have shown beneficial responses to PARPi, and recent clinical trials in PDAC have been performed to assess the beneficial role of second-line olaparib monotherapy in *BRCA1/2*-deficient patients, following failure on gemcitabine.^177^ PARPi work on the basis of synthetic lethality whereby tumours with defects in double-stranded DNA repair pathways become dependent on PARP to repair the resultant collapsed replication forks and maintain chromosomal stability and cell cycle progression.^178–180^ Another option for patients with mutations in DNA repair pathways is by causing further DNA damage in these defective cells by either platinum-based therapies or mitomycin C.^56 181^ Furthermore, the PI3K pathway has a well-established role in DNA damage repair, and promising combination therapies in endometrial and breast cancers have motivated clinical trials in PDAC to assess the effect of PARPi in combination with PI3K pathway inhibitors (table 2, figure 3).^182–184^ The clear responses seen in patients presenting with these DNA repair defects provides a promising personalised approach to therapy when standard of care is found to be ineffective.

### Sensitisation of cell cycle defective tumours to cyclin-dependent kinase (CDK) inhibitors as combination therapy

Mutations in *CDKN2A*, *CCND1* and/or *CDK4/6* commonly occur in PDAC, and recent work has demonstrated that the reliance of some tumours on this pathway may sensitise them to CDK inhibitors (CDKi).^50^ In recent work, the sensitivity of a panel of PDCLs was assessed for their response to a CDKi, which identified PDCLs with high expression of retinoblastoma protein and low expression of p16^INK4A^ were significantly correlated with improved response to CDKi, in combination with gemcitabine.^185^ This is in line with work in breast and ovarian cancers, and melanoma, where this same expression pattern is common.^186–188^ Furthermore, it has been shown that in PDAC and other cancers, combinations of CDKi with PI3K pathway inhibition in subsets of patients can have an even greater response, thus stratification in this setting may warrant further investigation (table 2).^189 190^

### Histone deacetylases (HDACs) and mutant p53 inhibitors

Loss or mutation of the tumour suppressor p53 occurs in ~75% of patients with PDAC, where gain-of-function mutations occur at a higher prevalence and are thought to provide a growth advantage, as well as driving metastatic progression.^50 191 192^ The primary role of p53 is to bind DNA as a transcriptional activator or repressor, mediating transcriptional networks responsible for cell death and replicative senescence in response to genotoxic or oncogenic stress.^193 194^ HDACs work by regulating gene expression at an epigenetic level and have been associated with upregulation of mutant p53 in several cancers, including PDAC.^195 196^ Furthermore, several HDACs are overexpressed in PDAC, prompting assessment of the clinical benefit of their inhibition.^197 198^ Recently, a phase I clinical trial of vorinostat with chemoradiation in PDAC showed promising overall survival benefits.^199^ In parallel, emerging studies in other AdvSTs demonstrated promising synergistic benefits when combining vorinostat with the broad-spectrum RTKi sorafenib^200 201^ and subsequently led to a new phase I trial of vorinostat and sorafenib with chemotherapy in PDAC (NCT02349867).

One of the key mediators of p53 protein stability is mouse double minute 2 (MDM2), which is responsible for ubiquitination and subsequent degradation of p53 by the proteasome.^202^ Mutation of *CDKN2A* occurs in 35% of PDAC tumours leading to loss of expression of the tumour suppressors p16^INK4a^ and p14^ARF^ (p19^ARF^ in murine tumours).^50^ ARF inhibits MDM2, and hence loss of this tumour suppressor leads to increased levels of MDM2 and a decrease in p53 pathway activity.^203^ Another key mediator of MDM2 activation is Akt, which activates MDM2 in parallel with other survival pathways (figure 3).^61^ Furthermore, the tumour suppressor PTEN has been shown to bypass MDM2 and stabilise p53 protein levels, leading to downstream activity.^204 205^ In the complex mutational landscape of PDAC, it is unclear how PI3K pathway inhibitors may affect the already overexpressed levels of mutant p53.^206^ However, ~26% of PDAC tumours retain wild-type p53, and recent work demonstrated that MDM2 inhibitors were able to reactivate wild-type p53 pathway signalling in pancreatic cancer.^207^ This may suggest that PI3K pathway inhibition in PDAC could reduce MDM2 levels and facilitate activation of the tumour-suppressive functions of p53 and lead to apoptosis.^208 209^ Caution may need to be taken however, for patients with p53 mutations where upregulation of this mutant protein may enhance tumourigenesis.^210^ The complex relationship between these pathways includes GSK-3β, which has been implicated in MDM2 activation and p53 degradation.^211 212^ GSK-3β is a canonical Akt substrate and is inactivated by this phosphorylation interaction (figure 3).^61^ This complex regulation of MDM2 by different members of the PI3K pathway may explain the complex responses of p53 wild-type or deficient tumours when applying PI3K pathway inhibitors as radiosensitising agents.^213–216^

### Development of small molecule inhibitors for oncogenic *KRAS* and MAPK signalling

Mutations in *KRAS* occur in ~95% of PDAC cases, and this has prompted several efforts to target both mutant *KRAS* and the resultant aberrant downstream signalling.^26 38^ The predominant *KRAS* mutations in PDAC are *KRAS^G12D^* and *KRAS^G12V^*, where *KRAS ^G12D^* accounts for 83% of *KRAS* mutations in PDAC and has been shown to classify into more aggressive molecular subtypes.^41 217^ This aggressive classification may be due to the downstream signalling cascades that have been linked to specific *KRAS* mutants, where *KRAS^G12D^* predominantly activates the MAPK and PI3K pathways, whereas *KRAS^G12V^* predominantly activates Ral signalling.^218^ Several attempts have been made to inhibit oncogenic Ras isoforms by either competitive inhibition of GTP binding or by preventing membrane translocation but have so far failed to successfully inhibit Ras at a low enough dose for clinical efficacy.^217^ Similarly, the farnesyl transferase inhibitor, tipifarnib, did not prolong overall survival compared with gemcitabine alone in advanced PDAC.^219^ Interestingly, RNA interference approaches have been efficaciously applied to PDAC tumours that were metabolically reprogrammed by mutant Ras, where inhibition of the mutant isoform was sufficient to delay tumour growth.^220–222^ With this in mind and by successfully delivering small inhibitory RNAs (siRNAs), one group was able to demonstrate the in vivo application of a miniature biodegradable polymeric matrix for delivery of a *KRAS^G12D^*-targeted siRNA.^221^ Knockdown of oncogenic *KRAS^G12D^* at the transcript level effectively inhibited downstream pathways and reduced in vivo tumour burden. Another recent approach to deliver siRNAs against oncogenic *KRAS^G12D^* to PDAC tumours is using fibroblast-derived exosomes, termed iExosomes, which maintain CD47 expression and hence show increased bioavailability and tumour uptake.^220^

As an alternative approach to inhibition of oncogenic *KRAS*, innumerable inhibitors have been developed to target the key signalling cascades immediately downstream, namely the MAPK and PI3K pathways. Several clinical trials have been performed using MEKi in combination with gemcitabine,^223 224^ but these have so far failed to demonstrate significant improvements in survival, compared with gemcitabine alone. Inhibition of the MAPK pathway is regularly associated with an increase in PI3K pathway activity.^225 226^ Hence, new treatment strategies have emerged that aim at inhibiting both of these key effector pathways (table 2). While these follow from promising preclinical studies, where the combined efficacy of dual MAPK and PI3K pathway inhibition provides significant tumour growth inhibition, the combined toxicity of this approach can present a strong limiting factor.^226 227^ Notably, the sequential effect of targeting these pathways may increase tumour susceptibility to inhibition while potentially minimising toxicity.^228^

## Microenvironmental influences on drug response

**Figure 4:**
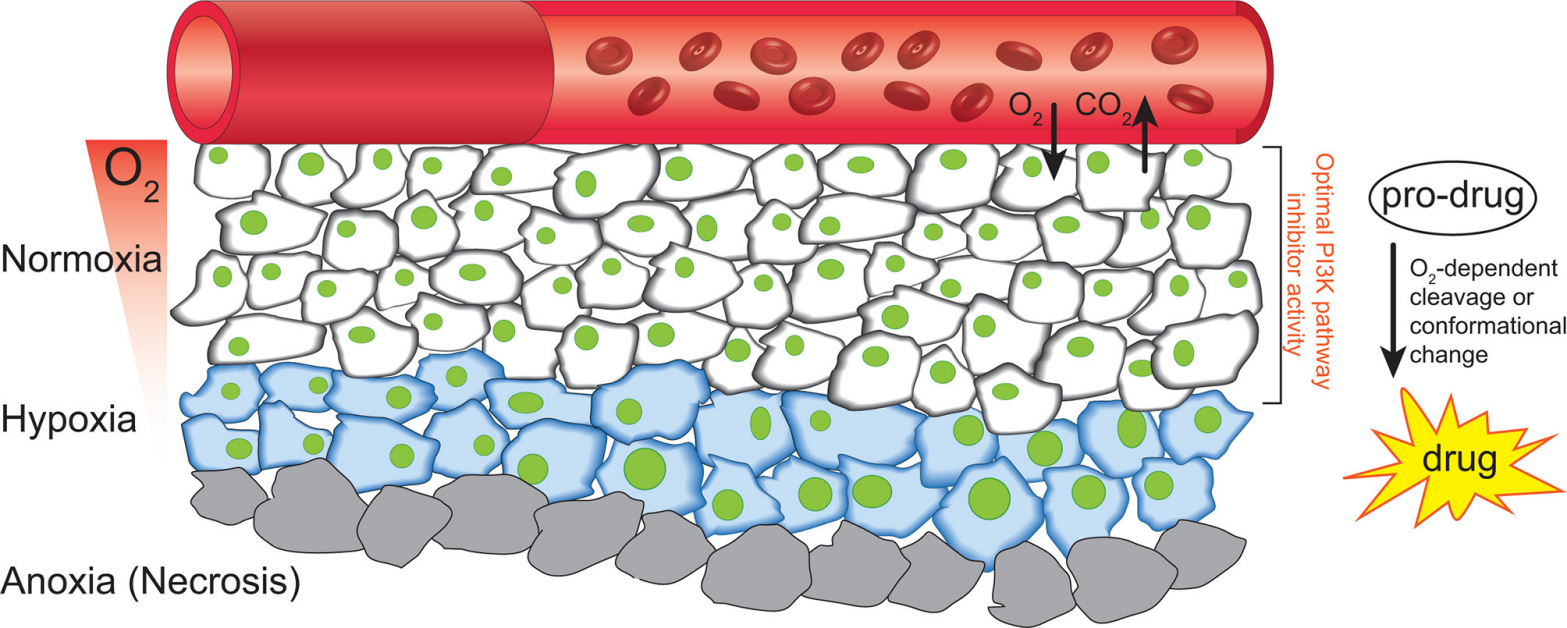
Schematic of the formation of a hypoxic environment and the potential targeting of this microenvironment with HAPs. RBCs transport oxygen through the blood vasculature, and hypoxia forms when this diffusion-limited process delivers insufficient oxygen to cells distant to the vasculature (blue cells). The extreme case of anoxia (grey cells) regularly results in necrotic cell death. HAPs take advantage of the hypoxic environment of tumours to deliver cytotoxic compounds to these tumour regions, where the prodrug is either enzymatically cleaved by the cells metabolic machinery or undergoes a conformational change in response to the low oxygen partial pressure. HAPs, hypoxia-activated prodrugs; RBCs, red blood cells.

PDAC is characterised by a highly desmoplastic reaction, commonly associated with high levels of stromal infiltration, ECM deposition and tumour hypoxia.^47 229^ Targeting of the ECM or associated stroma has shown some efficacy in PDAC, by improving drug delivery and sensitising tumours to chemotherapy,^230–232^ although the viability of targeting the stroma in PDAC remains controversial. Complete stromal ablation in PDAC was shown to enhance cancer aggressiveness,^233 234^ which calls for more subtle and targeted approaches to normalising instead of completely ablating the tumour-associated stroma.^47 235^ This effect is partly thought to occur due to a normalisation of the tumour vascular network and manipulation of the ECM/stroma, improving drug efficacy in the tumour.^231 235 236^ Another common feature resultant from enhanced desmoplasia is the development of a hypoxic environment (figure 4). Hypoxia is strongly associated with increased radioresistance, chemoresistance and metastasis,^237–239^ and PDAC is among those cancers with a propensity for high levels of tumour hypoxia, which is predictive of poorer patient prognosis.^229 240^

Reduced oxygen consumption and increased glycolysis were recently identified by mitochondrial genome sequencing in PDAC PDCLs.^241^ This is a key aspect of the Warburg effect, which predicts tumours to rely more heavily on glycolysis for their metabolism.^242^ While this presents an advantage for tumours that experience reduced vascularity and oxygen levels, it also presents an opportunity to potentially starve the tumour in these hypoxic regions.^243^ One of the first steps for tumours to switch to glycolytic metabolism is an increase in lactate dehydrogenase (LDH) activity, which converts pyruvate into lactate.^244^ Inhibition of LDH has recently been shown to synergise with gemcitabine in vitro and may provide a novel strategy for PDAC.^245^ Another important aspect of PDAC metabolism is the metabolic reprogramming resultant from *KRAS* mutation, which upregulates glucose uptake and biomass synthesis.^246^ Furthermore, the upregulation of MUC1 during PDAC progression, along with HIF1α in hypoxic tumour regions, has been shown to cooperate by upregulating anabolic metabolism through the pentose phosphate pathway, resulting in gemcitabine resistance.^247^ The glycolytic switch in hypoxia can lead to a decrease in pH, and hence pH-regulating proteins are also an important downstream target of the cellular hypoxic response.^248^ Inhibitors of these pH-regulatory components are currently being assessed, after promising preclinical work, for their role in limiting tumour growth.^249 250^ The PI3K pathway also plays an important role in glucose uptake, amino acid metabolism and response to cellular stress.^58 60 61^ In hypoxia, Akt activity is upregulated, along with glucose transporters, to facilitate the switch to anaerobic metabolism.^105 214 238 243^ Moreover, treatment of PDAC with PI3K pathway inhibitors is less effective in hypoxia, highlighting an important microenvironmental consideration for future stratified clinical trials.^105 251^

With this in mind, we recently demonstrated that a hypoxia-activated prodrug (HAP) could alleviate hypoxia-induced resistance to a PI3K pathway inhibitor in a combination therapy.^105^ HAPs are typically metabolised by enzymatic reduction in hypoxia from a primarily inactive form to an active form while having limited effects on normoxic or healthy tissues, making them ideal for development of combination therapies (figure 4; reviewed in ref 238). This approach was also found to be effective in renal cell carcinoma, where combination with rapalogs and the HAP TH-302 significantly improved survival in preclinical models.^252^ The efficacy of these HAPs began to be realised with tirapazamine, which showed a high differential toxicity in hypoxia, compared with normoxia, which made it an ideal candidate for combination with radiotherapy.^253 254^ However, despite promising phase II clinical trials in squamous cell carcinoma, a phase III trial failed to show improved efficacy over chemoradiotherapy alone.^255^ Notably, this trial was not biomarker driven and hence did not stratify patients based on hypoxic tumour burden, which may have disguised any potential efficacy in a subset of patients. This is a common issue in clinical trials and is also implicated in the phase III failure of TH-302 after promising phase II results (NCT01746979).^256^ The preclinical promise and potential applications of HAPs in combination therapies means that despite the phase III setbacks, improved tirapazamine analogues, such as SN3000 and SN29751, as well as other recently developed HAPs, including PR-104 and AQ4N, are all under preclinical/clinical investigation.^257–259^

Recent work by our group demonstrated that a hypoxic gene signature was associated with a poorer prognosis for patients with PDAC.^229^ While this is a promising approach, application in the clinic may be limited by the collection of patient biopsies, which may not adequately represent the extent of hypoxia within the whole tumour. The necessary stratification of patients for future clinical trials calls for a method to assess the whole tumour non-invasively (figure 2). To this end, positron emission tomography approaches have been developed based on radiolabelled 2-nitroimidazoles or antibodies, which can be coupled with ^18^F–fluorodeoxyglucose imaging to first identify malignant lesions.^238^ Similarly, non-invasive imaging of oxygen partial pressure using electron paramagnetic resonance imaging or assessment of pyruvate metabolism by MRI have also been used to stratify PDAC tumours for treatment with TH-302 and radiation.^260^

Moving forward, the design and synthesis of HAPs with defined molecular targets are emerging for specific applications. For example, hypoxia-activated chk1 inhibitors were recently developed as proof-of-principle molecules for targeting the hypoxic compartment of tumours, where chk1 is an important component of the DNA damage response and cell cycle progression.^261^ From these studies, it is clear that the emerging application of microenvironmental-targeted agents in combination therapies can improve patient outcomes, and as newer generation inhibitors are developed, we are likely to see a wider application of these agents entering the clinic.

## Conclusions

Given the lagging improvements in therapy, there is a dire need to find new biomarkers and targets to move pancreatic cancer towards personalised medicine approaches (figure 2). To guide clinical success, emerging combinations would benefit from a preclinical platform of evidence in at least one in vivo model, as well as optimisation of solubility for reduced toxicity and, importantly, identification of at least one suitable biomarker for patient stratification at the level of clinical trials (figure 2). The emerging efficacy of PI3K pathway inhibitors for PDAC and the convergence of several aberrantly expressed signalling cascades highlights a clear progression towards their application for this disease. For example, patients with aberrant DNA damage repair pathways have responded well to PI3K pathway inhibition as part of combination therapies, and trials are already underway in PDAC. Furthermore, given the complex dimerisation of the ErbB family of RTKs and the association of Ephrin receptors with more aggressive PDAC subtypes, RTKs may provide biomarkers for patients that would respond efficaciously to PI3K pathway inhibition. Moving forward, one of the key goals of the ICGC2 is to link bioinformatics approaches, such as molecular subtyping of patients, to clinical data, and we expect this to drive an increase in biomarker-driven clinical trials (proposed in box 2). This is a necessary step to decrease the attrition of lead compounds in the pharmaceutical industry and to ensure that next-generation inhibitors progress to patients that are appropriately subtyped for maximum benefit.

Box 2 **What may improve clinical trials?**

- Patient subtyping from tumour biopsies by genomic and/or mass spectrometry approaches.
- Biomarker identification prior to progression to phase II/III studies to ensure appropriate patient stratification for maximal benefit (circulating cell-free DNA/genomic approaches/IHC).
- Incorporation of non-invasive imaging for hypoxic tumour burden, such as electron paramagnetic resonance imaging, MRI or positron emission tomography with ^18^F-fluorodeoxyglucose.
- Testing of promising lead compounds against stratified patient-derived xenograft/Avatar cohorts prior to phase I clinical trials.
- Development of new prodrugs to use in combination therapies with reduced off-target effects.
- Raising the bar when defining preclinical ‘success’.
